# Glioma grade and post-neurosurgical meningitis risk

**DOI:** 10.1007/s00701-024-06193-w

**Published:** 2024-07-18

**Authors:** Sakke Niemelä, Jarmo Oksi, Jussi Jero, Eliisa Löyttyniemi, Melissa Rahi, Jaakko Rinne, Jussi P. Posti, Dan Laukka

**Affiliations:** 1 Department of Otorhinolaryngology, Turku University Hospital and University of Turku, Turku, Finland; 2 Department of Infectious Diseases, Turku University Hospital and University of Turku, Turku, Finland; 3 Department of Otorhinolaryngology, Head and Neck Surgery, Helsinki University Hospital and University of Helsinki, Helsinki, Finland; 4 Unit of Biostatistics, University of Turku and Turku University Hospital, Turku, Finland; 5 Clinical Neurosciences, University of Turku, Turku, Finland; 6 Department of Neurosurgery, Neurocenter, Turku University Hospital, Turku, Finland

**Keywords:** Bacterial meningitis, Complication, Post-neurosurgical meningitis, Glioma surgery

## Abstract

**Background:**

Post-neurosurgical meningitis (PNM) constitutes a grave complication associated with substantial morbidity and mortality. This study aimed to determine the risk factors predisposing patients to PNM following surgery for low- and high-grade gliomas.

**Methods:**

We conducted a retrospective analysis encompassing all patients who underwent glioma surgery involving craniotomy at Turku University Hospital, Turku, Finland, between 2011 and 2018. Inclusion criteria for PNM were defined as follows: (1) Positive cerebrospinal fluid (CSF) culture, (2) CSF leukocyte count ≥ 250 × 10^6^/L with granulocyte percentage ≥ 50%, or (3) CSF lactate concentration ≥ 4 mmol/L, detected after glioma surgery. Glioma grades 3–4 were classified as high-grade (n = 261), while grades 1–2 were designated as low-grade (n = 84).

**Results:**

Among the 345 patients included in this study, PNM developed in 7% (n = 25) of cases. The median time interval between glioma surgery and diagnosis of PNM was 12 days. Positive CSF cultures were observed in 7 (28%) PNM cases, with identified pathogens encompassing *Staphylococcus epidermidis* (3), *Staphylococcus aureus* (2), *Enterobacter cloacae* (1), and *Pseudomonas aeruginosa* (1). The PNM group exhibited a higher incidence of reoperations (52% vs. 18%, p < 0.001) and revision surgery (40% vs. 6%, p < 0.001) in comparison to patients without PNM. Multivariable analysis revealed that reoperation (OR 2.63, 95% CI 1.04–6.67) and revision surgery (OR 7.08, 95% CI 2.55–19.70) were significantly associated with PNM, while glioma grade (high-grade vs. low-grade glioma, OR 0.81, 95% CI 0.30–2.22) showed no significant association.

**Conclusions:**

The PNM rate following glioma surgery was 7%. Patients requiring reoperation and revision surgery were at elevated risk for PNM. Glioma grade did not exhibit a direct link with PNM; however, the presence of low-grade gliomas may indirectly heighten the PNM risk through an increased likelihood of future reoperations. These findings underscore the importance of meticulous post-operative care and infection prevention measures in glioma surgeries.

## Introduction

Gliomas are serious medical conditions consisting of approximately 80% of all malignant brain tumors [32]. The incidence rate of gliomas is around 7/100 000 in Finland [25]. High-grade gliomas (grade 3–4) constitute 85% of all gliomas while the rest are low grade gliomas (grade 1–2) [27]. Five-year survival rate for high-grade gliomas ranges from 3 to 22% and for low grade gliomas from 54 to 82% [27].

The incidence of surgical site infections following craniotomy ranges from 1 to 10%, while the occurrence of post-neurosurgical meningitis (PNM) varies between 2%-9% [16, 20, 29]. Approximately 3% of patients undergoing glioma surgery necessitate reoperations due to post-operative infections [22]. PNM causes clinically significant morbidity and mortality and prolongs treatment periods and causes potentially adverse effects regarding patient survival [13, 16, 29, 31].

Risk factors for PNM include reoperations, placement of postoperative ventricular shunts and lumbar catheters, male sex, diabetes mellitus, corticosteroid use, cerebrospinal fluid (CSF) leak, prolonged surgery, and genetic predisposition [1, 16, 17, 19, 20, 29]. Gliomas are associated with immunosuppression while immune profiles may vary between different glioma grades [3, 14]. Therefore, glioma grade might affect perioperative risk of infection.

Prophylactic antibiotics have been shown to reduce superficial and deep infections during neurosurgery [17], however, some studies prefer the correct sewing technique over antibiotic prophylaxis to prevent postoperative infections as the prevention of CSF leak is of utmost importance to prevent [4]. The recommended regimen is targeted mainly against gram-positive agents, such as *Staphylococcus epidermidis* and *Staphylococcus aureus*, which have been the main pathogens causing PNM [30]. Perhaps due to this selection of prophylactic antibiotics, gram-negative bacteria such as *Acinetobacter baumannii, Klebsiella pneumoniae* and *Escherichia coli* have become increasingly common agents causing PNM according to recent studies [18, 34].

Careful practices during suture sewing are essential to prevent infections. Deep infections often require surgical debridement or reoperation along with systemic antibiotics to overcome the infection [17]. Along with gold-standard CSF culture for detection of pathogens, imaging with magnetic resonance imaging of suspected PNM patients is often required to rule out abscess or empyema, and to possibly detect hydrocephalus [16].

While many previous studies have focused on surgical site infections following glioma surgery, the incidence of PNM and its associated risk factors remain uncertain. It is also unclear whether the grade of glioma impacts the risk of PNM, as most studies have included only high-grade gliomas. Implementing effective infection prevention strategies and vigilant surveillance for post-operative infections are essential to optimize outcomes in this patient population.

Our aim was to investigate the incidence of PNM after surgery for gliomas of different grades, and risk factors associating with PNM in Turku University Hospital, Finland, between 2011–2018.

## Methods

### Patient selection

This was a retrospective, descriptive cohort study. Data collection was retrolective. A database search for glioma patients treated with craniotomies between 2011–2018 in Turku University Hospital, Finland, was performed. Our institution serves as a tertiary referral center within the Hospital District of Southwest Finland, overseeing the management of all central nervous tumors among a population of approximately 490 000.

The investigation assessed patients who underwent craniotomy for World Health Organization grade 1–4 glioma. We systematically examined the pathological reports of a consecutive cohort comprising 1 161 individuals undergoing craniotomy for intracranial tumors between 2011 and 2018. International Classification of Diseases 10th edition codes C71.*, The Nordic Medico-Statistical Committee codes AAB00, AAB10 and AW*** were used to identify appropriate patients. Out of this cohort, 345 patients received a glioma diagnosis and were subsequently enrolled in the study. The patients operated for gliomas were defined as the intervention group.

Included patients for PNM had at least one of the possible symptoms of PNM defined as either fever, headache, decreased mental status, seizures, or neck stiffness. Additionally, they had to have at least one of the following inclusion criteria: 1. CSF culture positivity, 2. CSF leukocyte count ≥ 250 × 10^6^/L with granulocyte percentage ≥ 50% [15] or 3. CSF lactate ≥ 4 mmol/L [16]. Finally, 25 patients with PNM were identified. CSF samples were usually obtained by lumbar puncture after imaging of the head showed that there was no evidence of increased intracranial pressure and laboratory results showed that there were no abnormalities in blood coagulation. Occasionally, a CSF sample was obtained from a craniotomy wound site on admission following the safety measures described above. Outcome was classified into either patients who developed PNM (cases) and patients without PNM (controls).

### Definitions

Revision surgery was defined as a reoperation occurring earlier than 100 days after primary surgery, and reoperation was defined as a repetitive glioma surgery in the patient history without time limits. The revision and reoperation surgeries were performed from the same site as the index surgery.

The reason for reoperations were confirmed or suspected cases of recurring or residue gliomas after imaging assessment. The reasons for revision surgeries were any complication requiring revision surgery such as hydrocephalus, CSF leak from the wound or surgical site infection or intracranial infections.

The suspect of a local wound infection rose if the wound had swelling, redness, pain, or secretion of pus.

### Preoperative management

The skin of operative area is cleaned with chlorhexidine. Patients received routinely cefuroxime 3 g intravenous infusion in the operation room before the start of surgery. Patients not tolerating cefuroxime, intravenous clindamycin 600 mg was used as prophylaxis instead. Patients did not routinely receive any follow-up antibiotics. All patients undergoing revision surgery or reoperations received the above stated antibiotic prophylaxis. The skin is closed subcutaneously and intradermally with antibacterial sutures [23] and the skin is closed with clips. The techniques for sterilization of the scalp, the type and duration of prophylactic antibiotics were similar in all aspects for low-grade glioma surgery and high-grade glioma surgery. The only exception is reoperations, which are more common in low-grade glioma patients and for which some neurosurgeons advocate the use of postoperative antibiotics for 24–48 h.

### Statistical analysis

Distributions were evaluated using visual evaluation, normal quantile plot, skewness and kurtosis measures. Due to the skewness of the distributions, we used medians in reporting results. Continuous data were summarized with median and lower (Q1) and upper (Q3) quartile and compared between patients with PNM and those without PNM with Wilcoxon rank sum test. Association between two categorical variables was first evaluated with Fisher’s exact test. Furthermore, logistic regression was performed to reoperation and revision surgery, separately for each explanatory variable (univariate approach) and reported with odds ratio (OR) and its 95% confidence intervals (CI). After univariate modelling, multivariable model was built up based on univariate results. P values of < 0.05 (two-tailed) were regarded as statistically significant. The data analysis was generated using SAS software, Version 9.4 of the SAS System for Windows (SAS Institute Inc., Cary, NC, USA).

### Data availability statement

The data generated during this study is available from the corresponding author on a reasonable request.

## Results

### Background information and univariate analysis

Out of the 345 patients who underwent glioma surgery, the median age was 60 years (Q1-Q3: 44–68 years), with 46% (n = 159) being female. High-grade glioma was diagnosed in 76% of cases, while low-grade glioma was diagnosed in 24% of cases, and 20% of the surgeries were reoperations due to recurrent glioma.

PNM rate was 7% (n = 25), with a median time interval between glioma surgery and the diagnosis of PNM being 12 days (Q1-Q3: 7–20 days). Patients who sustained PNM were significantly younger, with a median age of 49 years (Q1-Q3: 32–62 years) compared to 60 years (Q1-Q3: 46–68 years) in those without PNM (p = 0.013). Additionally, patients with PNM had low-grade gliomas 44% (11/25) compared to high-grade gliomas 56% (14/25), p = 0.027. However, with low-grade gliomas, PNM was occurring more often (13%, 11/84) versus high-grade (5.4%, 14/261). Patients with PNM had a greater frequency of reoperations (52% vs. 18%, p < 0.001) and revision surgeries (40% vs. 6%, p < 0.001) compared to those without PNM. Statistically significant associations between gender and reoperation (p = 0.35), revision surgery (p = 0.43) or low-grade/high-grade gliomas (p = 0.45) were not found. Other statistical analyses shown in Table 1.

**Table 1:** Univariate Analysis of Risk Factors for post-neurosurgical meningitis (PNM)

VARIABLE	PNM (n = 25)	No PNM (n = 320)	p-value
Age, median (Q1-Q3)	49 (32–62)	60 (46–68)	0.01
Sex (female)	14 (56%)	145 (45%)	0.31
Sex (male)	11 (44%)	175 (55%)	0.31
BMI, median (Q1-Q3)	28 (23–31)	26 (24–30)	0.54
ASA classification			0.97
ASA 1	0 (0%)	4 (1%)	
ASA 2	2 (8%)	27 (8%)	
ASA 3	15 (60%)	197 (62%)	
ASA 4	8 (32%)	87 (27%)	
ASA 5	0 (0%)	1 (0%)	
Glioma grade			0.03
Grade 1	4 (16%)	13 (4%)	
Grade 2	7 (28%)	60 (19%)	
Grade 3	3 (12%)	41 (13%)	
Grade 4	11 (44%)	206 (64%)	
High-grade vs. Low-grade glioma			0.027
Low-grade (grade 1–2)	11 (44%)	73 (23%)	
High-grade (grade 3–4)	14 (56%)	247 (77%)	
Reoperation	13 (52%)	57 (18%)	0.001
Revision surgery	10 (40%)	18 (6%)	0.001
Emergency surgery	3 (12%)	11 (3%)	0.07
Operation time (min), median (Q1-Q3)	202 (165–301)	207 (156–251)	0.22
CSF sample available	25 (100%)	24 (8%)	0.001
CSF leukocyte count (cells/mm^3^), median (Q1-Q3)	425 (86–1140)	7 (1–35)	0.001
CSF neutrophil count (%), median (Q1-Q3)	79 (55–92)	2 (0–9)	0.001

***ASA*** American Society of Anesthesiologists, ***BMI*** body mass index,, ***CSF*** cerebrospinal fluid, ***PNM*** post-neurosurgical meningitis, ***Q1*** lower quartile, ***Q3*** upper quartile

Nearly all patients who had received previous radiotherapy also underwent re-operation. Due to this significant overlap, we determined that including both factors in the same analysis would not be logical, as it could confound the results. Additionally, we noted that re-operations were more frequent than previous radiotherapy in the PNM group.

### Multivariable analysis

Multivariable analysis revealed that reoperation (OR 2.63, 95% CI 1.04–6.67) and revision surgery (OR 7.08, 95% CI 2.55–19.70) were significantly associated with PNM, while age (OR 0.98, 95% CI 0.95–1.01) and glioma grade (high-grade vs. low-grade, OR 0.81, 95% CI 0.30–2.22) showed no significant associations (Table 2).

**Table 2:** Results of multivariable analysis

Covariate	OR (95% CI)	P-value
Age	0.98 (0.95–1.0)	0.11
Glioma grade (grade 3–4 vs. grade 1–2)	0.81 (0.30–2.2)	0.69
Revision surgery	7.1 (2.6–19.7)	< 0.001
Reoperation	2.6 (1.0–6.7)	0.042

***CI*** Confidence interval, ***OR*** Odds ratio

### Causative pathogens

**Fig. 1:**
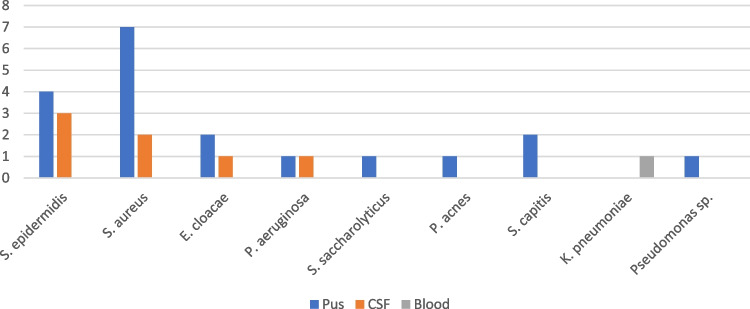
All bacteria detected from 345 patients after glioma surgery. Y-axis shows the number of bacteria

Positive CSF cultures were observed in 7 (28%) PNM cases, The pathogens acquired from CSF culture were *S. epidermidis* (3), *S. aureus* (2), *Enterobacter cloacae* (1) and *Pseudomonas aeruginosa* (1). There were 40 (12%) patients with suspected wound infection, of which 19 (6%) presented pathogen in bacterial culture causing the local infection. Five patients with culture confirmed local wound infection also had CSF culture confirmed PNM. The difference was not statistically significant (p = 0.1). Blood cultures were taken from 58 (17%) of total patients with only one (0.3%) blood culture positive patient. All pathogens detected are presented in Fig. 1.

## Discussion

This study retrospectively analyzed the characteristics of PNM occurring after glioma surgery at Turku University Hospital, Finland, between 2011–2018. The incidence of PNM after glioma surgery was 7%. The grade of glioma was not an independent risk factor for PNM, but low-grade gliomas had a higher rate of PNM because of the higher proportion of reoperations. Reoperation and revision surgery for post-operative wound complications were independent risk factors for PNM.

Patients with high-grade gliomas are older and thus have more comorbidities than their younger counterparts [25]. Therefore, it would be plausible that patients with high-grade gliomas would be more prone for PNM than patients with low-grade gliomas. Surprisingly, our study found that the PNM rate was higher in low-grade gliomas when compared to high-grade gliomas, as it would be reasonable to think the other way around, because for example operation time is usually longer considering high-grade gliomas, and thus the risk of postoperative infections could be higher.

However, in the multivariable analysis, glioma grade did not reach independent risk factor for PNM. Incidence of PNM was in the upper proportion when compared to previously reported incidences of 0.5%-8.9% [11, 19, 29]. The perioperative measures to prevent infections during craniotomy are in need of constant evaluation and improvements. A study by Cao et al. in 2017 found that prophylactic antibiotics did not prevent postoperative infections in clean craniotomies but caused more CSF negative cultures and more multidrug-resistant bacteria [4]. They concluded that precise sewing techniques may be even more important than antibiotic prophylaxis in the prevention of postoperative infections, for example to prevent CSF leakage, which is a major risk factor for developing PNM [4]. The role of diathermy compared to scalpel in regards on SSI were investigated in a Cochrane review, and no statistical differences were found, although the trials were underpowered [5, 23]. The overall careful tissue handling, and the use of antibacterial sutures is proven to reduce SSIs [23]. This is worth consideration, but antibiotic prophylaxis is nevertheless recommended [17, 31]. Using vancomycin intravenously combined with cephalosporins as prophylactic antibiotics during craniotomies seems to decrease the risk of surgical site infections [10]. Also, the use of vancomycin powder has been proven to reduce postoperative infections [28]. Routine CSF sampling among asymptomatic patients should be avoided [16].

A study performed in 2009 found that there was no statistically significant difference on survival between patients with or without postoperative infections after glioma surgery [2]. A few years after, a study with contradictory findings by De Bonis et al. presented that postoperative infections may in fact prolong the survival of patients (medians 16 months vs. 30 months) after glioblastoma surgery, perhaps by activating immune system [12]. However, another study found that glioma patients with postoperative infections tend to have 30% decreased median overall survival [31]. Even more recently, a study with a large population of 3784 patients found that there was no statistically significant difference on survival between patients with or without postoperative infections (median 5 months vs. 6 months, p = 0.17) [8]. PNM after glioma surgery often leads to delays in postoperative treatments such as radiotherapy or chemotherapy [31]. PNM also causes prolongation of hospital stay and thus more costs [16, 22].

Reoperations are a significant risk factor for developing PNM after glioma surgery [7, 19, 22, 29]. Risks of reoperations should be noted and carefully considered with each patient’s medical history in mind. Frailty has been recognized as an indicator of unfavorable outcome, post-operative complications, and mortality among patients with glioblastoma [21, 35], but unfortunately, we were not able to implement frailty-index in our study. There were no statistical differences on developing PNM with ASA class or duration of the surgery, as previously reported in some studies [17], but another study presented that ASA class or glioma grade were not statistically associated with elevated risk for developing PNM [22]. Glioma grade does not seem to be associated with postoperative complications in general by a recent paper by Morshed et al. [24].

The demographic data of patients with and without PNM were quite similar, which is important to note in clinical work. We proved that PNM is a compartmentalized central nervous system infection, because a large part of blood cultures remained negative (98%).

The rate of detected CSF culture positivity of 28% was lower compared with previous studies [26, 29]. However, recently the incidence of CSF culture positive postoperative meningitis has been decreasing according to a study performed in China in 2014, where only 10% of patients had a CSF culture positive PNM after craniotomy [6]. The use of prophylactic antibiotics at the beginning of the surgery has been proven to be effective in prevention of postoperative superficial and deep infections [17] – however, pre-diagnostic antibiotics reduces the positivity rate of the standard CSF culture [9], but the strategy would nevertheless be supported. The use of multiplex or quantitative polymerase chain reaction has been proven to be more sensitive and specific methods detecting pathogens compared to CSF culture, which should be used more often in the future [9].

Emergency surgeries were relatively rare (4%) in patients undergoing craniotomy. The frequency of PNM was higher in patients undergoing emergency surgery, but there was no statistically significant difference compared to patients undergoing elective surgery (p = 0.07). Old age and emergency surgeries have been identified as risk factors for PNM in previous studies [7, 33]. However, in our study, younger age of patients seemed to controversially be a risk factor for PNM. That may be because younger patients undergo revision surgeries or reoperations more often, and therefore they are at higher risk of PNM. The reasons for the 7% incidence of PNM after glioma surgery could be due to careful case ascertainment, and furthermore, the background is complex and cannot be further investigated in the current study setting.

This study has some limitations. Being a single center study is a limitation, and secondly, the risk of inaccuracy in the retrospectively documented data of patients is always possible. We were not able to include imaging results of patients, which is also a limitation. All patients received perioperative antibiotic prophylaxis, but we were not able to gather information on the rate of antibiotic administration to treat PNM before lumbar puncture. It is possible that some culture-negative PNM cases may have been aseptic meningitis. However, the solid definition of inclusion of PNM patients was a strength in this study, and it probably ruled out aseptic meningitis cases. It is also possible, that the included patients may not represent the whole population of glioma surgery patients in Finland.

## Conclusions

This study found a 7% incidence of PNM after glioma surgery. CSF culture positivity rate was 28%. Reoperations and revision surgeries were independent risk factors for PNM. We found a higher rate of PNM in patients with low-grade than high-grade gliomas, possibly due to higher proportion of reoperations. Glioma grade was not an independent risk factor for PNM. Further research is needed in future to develop efficient strategies to prevent PNM complications by identifying risk factors.

## Abbreviations

ASA: American Society of Anesthesiologists
BMI: Body mass index
CI: Confidence interval
CSF: Cerebrospinal fluid
OR: Odds ratio
PNM: Post-neurosurgical meningitis
Q1: Lower quartile
Q3: Upper quartile
